# Electrostatic pull-in application in flexible devices: A review

**DOI:** 10.3762/bjnano.13.32

**Published:** 2022-04-12

**Authors:** Teng Cai, Yuming Fang, Yingli Fang, Ruozhou Li, Ying Yu, Mingyang Huang

**Affiliations:** 1 College of Electronic and Optical Engineering & College of Microelectronics, Nanjing University of Posts and Telecommunications, Nanjing, China; 2 National and Local Joint Engineering Laboratory of RF Integration and Micro-Assembly Technology, Nanjing University of Posts and Telecommunications, Nanjing, China

**Keywords:** electrostatics, MEMS, microfluidics, NEM switches, pull-in

## Abstract

The electrostatic pull-in effect is a common phenomenon and a key parameter in the design of microscale and nanoscale devices. Flexible electronic devices based on the pull-in effect have attracted increasing attention due to their unique ductility. This review summarizes nanoelectromechanical switches made by flexible materials and classifies and discusses their applications in, among others, radio frequency systems, microfluidic systems, and electrostatic discharge protection. It is supposed to give researchers a more comprehensive understanding of the pull-in phenomenon and the development of its applications. Also, the review is meant to provide a reference for engineers to design and optimize devices.

## Introduction

It has become more and more difficult for traditional electronic devices made of rigid substrates to meet the needs of flexible and low-cost applications in complex environments. Flexible electronics have great potential for applications such as portable displays, electronic skin, and wearable healthcare. With the development of new materials and microelectromechanical and nanoelectromechanical systems (MEMS/NEMS), MEMS devices have become an essential part of flexible electronic systems. Common flexible MEMS devices are based on electrostatic, piezoelectric, and thermal actuation. Electrostatic actuation is one of the earliest and most mature actuation mechanisms used in flexible MEMS devices. Because of its simple structure and convenience for integrated manufacturing, it is one of the most actively researched driving actuation [1].

There are typical pull-in phenomena in electrostatic MEMS devices. Previous research has established that the pull-in is a widely acknowledged instability condition that restricts the normal operating range of the devices. For instance, the pull-in between the combs can cause the leakage of the energy, which needs to be avoided in the design of microcombs [1]. Meanwhile, devices using pull-in as the working mechanism have attracted researcher’s attention because of the low cost and the simple structures.

Extensive research has shown that modeling the bending of the flexible devices is only limited to the mechanical behavior of the flexible interconnect lines, and hardly involves the structure of the flexible devices. Nanoelectromechanical (NEM) switches made of carbon nanotubes (CNTs) [2–4], graphene (GR) [5–7], nanowires (NWs) [8–10], and other flexible materials are the most basic devices for a variety of component and system level applications, such as low-loss switches, phase shifts, and relays.

In this paper, the state of the art of electrostatic pull-in phenomena in flexible devices is discussed, and the influence of different electrode structures in NEM switches is classified and discussed. In addition, the applications of NEM switches in radio frequency (RF), electrostatic discharge (ESD), microfluidic, physical unclonable function (PUF), and microscale energy devices are summarized in this review, which will help researchers to understand upcoming trends regarding the pull-in effect and help engineers to further optimize the devices.

## Review

### Mechanism of the pull-in effect

The pull-in effect is a common phenomenon occurring in magnetostatic actuators, dielectric elastomer actuators, and electrostatic actuators, which can cause failure [1]. Electrostatic pull-in is a nonlinear effect caused by intensive electromechanical coupling, a unique characteristic for MEMS/NEMS devices, which break down before the pull-in occurs on the macroscale.

**Figure 1:**
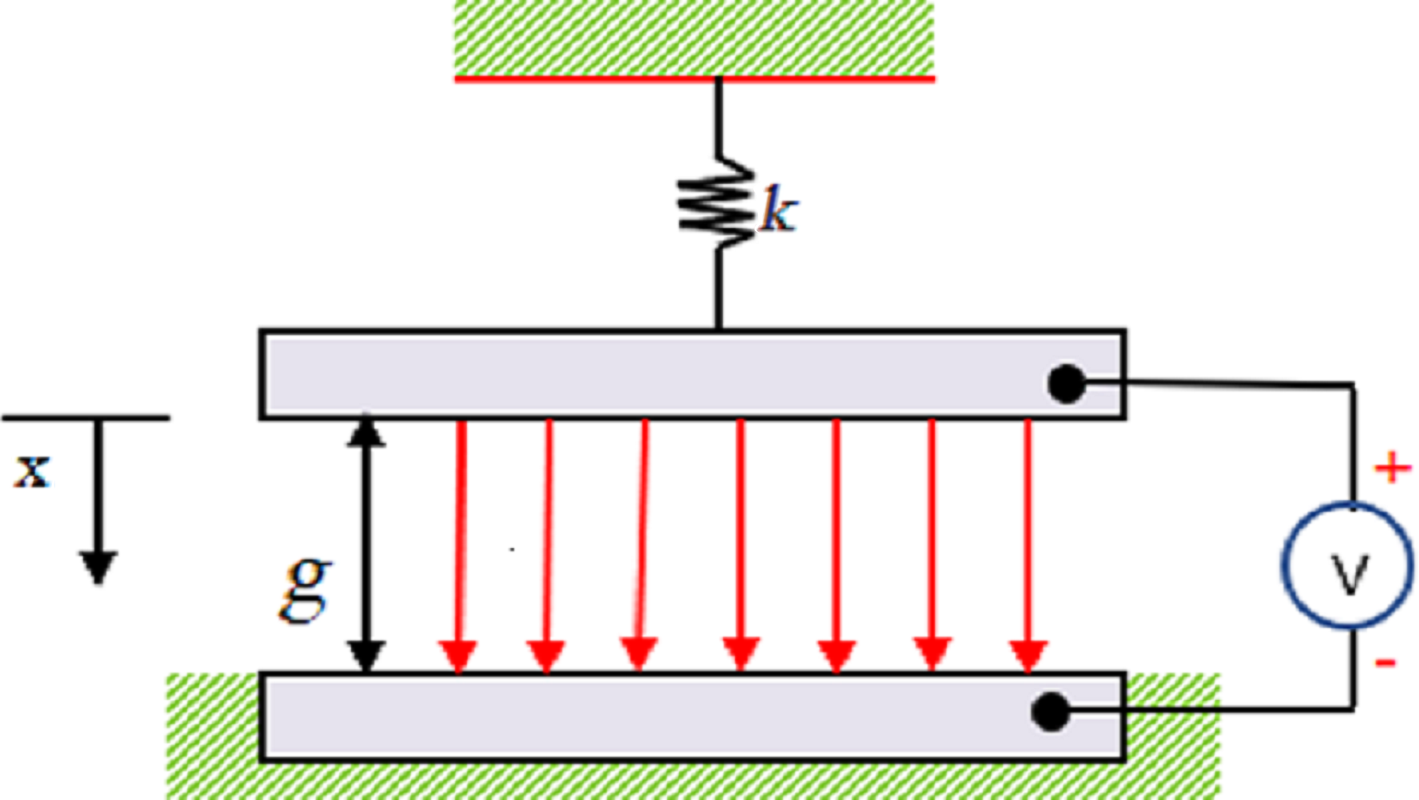
The lumped parameters model of the pull-in phenomenon between parallel plates. Figure 1 was redrawn from [11].

Figure 1 shows the lumped parameters model [11] of electrostatically driven parallel plate actuators. The structure consists of two parallel plates. The upper plate is suspended by a spring above the lower plate, which is fixed. The voltage between the plates creates an electrostatic force that causes the upper plate to move down, and the spring provides a restoring force that prevents it from moving. When the applied voltage is lower than the pull-in voltage, the electrostatic force and mechanical recovery force on the upper plate are balanced. As the voltage continues to increase, the upper plate moves rapidly downward and the two plates eventually contact, known as pull-in.

The critical voltage [11] that leads to the pull-in phenomenon is:


[1] $$V_{\text{Pull-in}}=\sqrt{\frac{8kg^{3}}{27\varepsilon_{0}A}},$$

where *k* is the elastic modulus of the flexible material, *g* is the initial distance between the two plates, ε_0_ is the vacuum permittivity, and *A* is the area of the two plates. It can be seen from Equation 1 that the material, size, and structure of the electrodes affect properties such as voltage, response time, and life cycles. When designing an ideal MEMS device, these parameters should be considered first.

### Electrostatic NEM switches

The simplest application of the pull-in phenomenon are NEM switches, which are the basis for the other component or system level applications. NEM switches represent a class of nanoscale devices, integrating both electrical and mechanical functionality of nanostructures to process external stimuli applied to the device controlling the electrical current [12]. The lower pull-in voltage and the improved durability of the NEM switches require electrode materials with high Young’s modulus, conductivity, and Poisson's ratio. The flexible suspension electrode materials include CNTs, GR, and silicon/germanium nanowires.

#### CNT-NEM switches

CNTs are an ideal flexible material for NEM electrodes because of the high aspect ratio, current density, and excellent tensile strength. In 2000, Rueckes et al. [13] observed the electromechanical conversion in CNTs for the first time. They used single-walled carbon nanotubes (SWCNTs) to prepare a suspended cross bistable switch array. Table 1 summarizes the structures and the voltage of CNT-NEM switches described in the literature. In addition to bridge and cantilever structures, the vertical structure further increases the device density and can be used in nonvolatile high-density memory devices. The properties such as diameter, length, morphology, and structure of CNTs directly affect the pull-in voltage and repeatability.

**Table 1:** Structures and pull-in voltages of CNT switches.

Ref	Structure	S/MCNT	Diameter [nm]	Length [nm]	Gap [nm]	Voltage [V]

[14]	bridge	M	20–40	800	40–60	3.6
[4,15–16]	bridge	S	1–3	130	20	3.5–4.5
[2]	bridge	S	—	—	—	2.5–3
[17]	cantilever	M	22	115	4	2.9
[18]	vertical	M	70	1400	100	20
[19–20]	vertical	M	50–70	—	100	4.5

**Figure 2:**
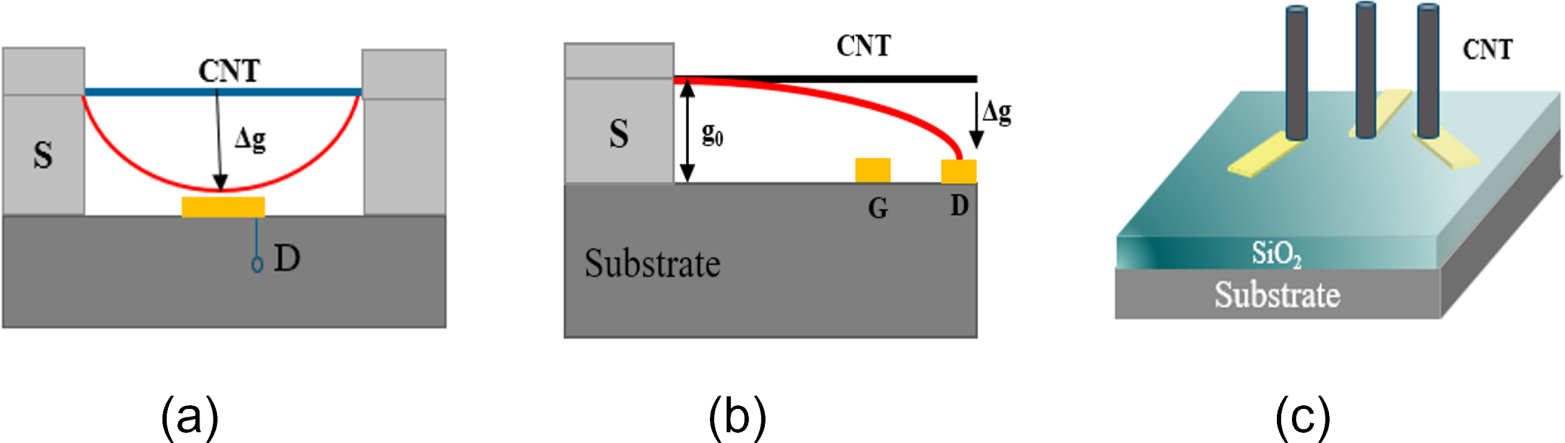
Structures of CNT switches. (a) Bridge model, (b) cantilever model, and (c) vertical model. Figure 2a,b,c were redrawn from [21], [22], and [18], respectively.

**Bridge model:** The typical structure of the bridge model is shown in Figure 2a. In 2006, Kaul et al. [15–16] prepared a two-terminal SWCNT NEM switch. The response time of the switch is nanoseconds, and the voltage is 3.5–4.5 V. Cha et al. [21] prepared a multiwalled carbon nanotube (MWCNT) NEM switch. The switch length is 800 nm, the diameter is 20–40 nm, the initial gap is 40–60 nm, and the critical voltage is about 3.6 V. Abbasi et al. [2] prepared a bistable switch based on SWCNTs, with a pull-in voltage of 2.5–3 V, which is suitable for nonvolatile memory applications.

**Cantilever model:** A typical cantilever structure is shown in Figure 2b. Lee et al. [22–23] prepared a three-terminal cantilever MWCNT NEM switch. The diameter of the CNTs is 50 nm, the length is 2 µm, and the gap is 150 nm. When *V*_ds_ is 0.5 V, *V*_gs_ is in the range of 6–30 V. Loh et al. [24] prepared a cantilever SWCNT and diamond electrode structure with a pull-in voltage of 23.4 V. C–C bonds can greatly improve the life cycles of the switch. The MWCNTs prepared by Dujardin et al. [17] had a diameter of 22 nm and a length of 115 nm. The pull-in voltage was reduced to 2.9 V as the gap was only 4 nm.

**Vertical model:** On the basis of the cantilever model, Jang et al. [18–20] and Kaul et al. [4] aligned CNTs vertically on the substrate. The vertically aligned MWCNTs were prepared by various methods, such as the constriction of CNT growth by nanoscale pockets and DC plasma-enhanced chemical vapor deposition (PECVD), as shown in Figure 2c. Using the vertically aligned CNT switches provides a high density for nonvolatile storage memory. However, vertically aligned switches require a pull-in voltage of more than 20 V, higher than the usual horizontally distributed switches.

CNT-NEM switches have great potential in nonvolatile memory devices. In addition, CNTs used with metals in composite electrodes, as a bottom contact electrodes [25–26], or intermediate layer [27], and can greatly improve the life cycles of switches. However, CNTs prepared by CVD are randomly oriented, and positioning CNTs at the desired location is a challenge that hinders scalable manufacturing. Ward et al. [28] and Cha et al. [21] used spin coating to disperse carbon nanotubes on a substrate by coating with a solution carbon nanotube powder in dichlorobenzene. In addition, a more general solution deposition process is AC dielectrophoretic technology [2,23]. The dielectrophoretic technology requires low voltage, high frequency, and can quickly realize positioning and alignment of CNTs in 10–60 s.

#### GR-NEM switches

Based on the large-scale fabrication of graphene (GR) using CVD and oxygen plasma etching, GR-NEM switches have attracted the attention of researchers. Table 2 summarizes GR-NEM switch structures described in the literature. Two common electrode materials are GR-Au and GR-GR. By adjusting the size of the graphene sheets, the number of layers and the gap, different pull-in voltages can be obtained.

**Table 2:** Structures and pull-in voltages of GR switches.

Ref	Structure	Length [µm]	Width [µm]	Thickness [layers]	Voltage [V]

[29]	GR-GR	20	3	1	5
[30]	GR-Au	1	4	2	4–5
[31]	GR-Au	1	2.5	2	5–7.5
[32]	GR-Au	1	–	20	10–12.5
[33–34]	GR-Au	2.5	0.5	2	2
[7,35]	GR-Au	1	2	1	1.92
[6]	GR-Au	1	2	10	3.8
[5]	GR-GR	1.5	0.8	1	6

**Figure 3:**
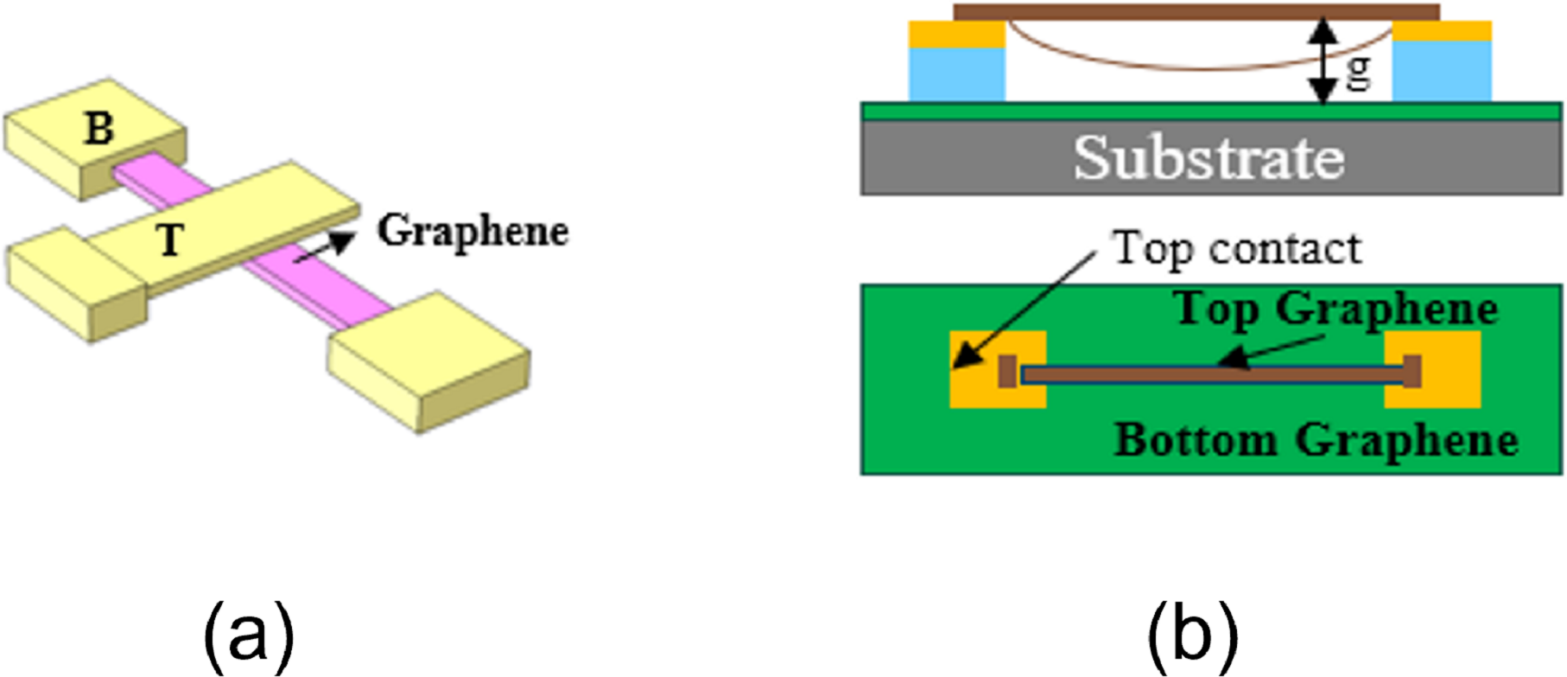
Structures of GR NEM switches. (a) GR-Au model and (b) GR-GR model. Figure 3a,b were redrawn from [7,29], respectively.

**GR-Au:** A typical GR-Au structure is shown in Figure 3a. As the structure requires a sacrificial layer to keep GR suspended and avoid collapse, the preparation technology is complex. Li et al. [30] prepared cantilever GR-NEM switches with two or three terminals using amorphous silicon as sacrificial layer. Sun et al. [6] prepared a three-terminal switch using PMMA polymer as the sacrifice layer. The graphene beam area was 2.5 µm × 0.5 µm, the gap was 170 nm, and the pull-in voltage was about 2 V. In 2016, Sun et al. [6] prepared a graphene cantilever switch with a thickness of about 10 layers, as shown in Figure 3a. The graphene sheet has an area of 1 µm × 2 µm, a gap of 1401 nm, and a critical voltage of 3.8 V. In addition, Liu et al. [34] proposed a GR-NEM switch structure with three-terminal circular clamping. The significant advantages of this geometry are the elimination of tearing and the limitation of stiction, providing a new direction for achieving reliable GR switches.

**GR-GR:**Figure 3b shows a typical GR-GR structure. Li et al. [33] used molecular dynamics to compare the adsorption energies of GR-Au and GR-GR structures. The GR-GR structure has a higher adsorption energy of 307 mJ/m^2^. Mizuta et al. [7,35] assumed that the use of a GR-GR electrode structure could avoid the uncontrollable microscale interactions between GR and the outer coating interface of the metal electrode. In addition, the use of a GR-GR electrode structure can improve the flexibility of the whole switch, laying a good foundation for fully flexible devices.

In 2009, Milaninia et al. [29] reported the first NEM switch with a GR-GR electrode structure. The bridge monolayer graphene electrode is 20 µm long and 3 µm wide, the electrode gap is 500 nm, and the pull-in voltage is less than 5 V. As the electrode material was polycrystalline graphene, fracture of the GR electrode was observed after 4–5 life cycles, resulting in device failure.

**Figure 4:**
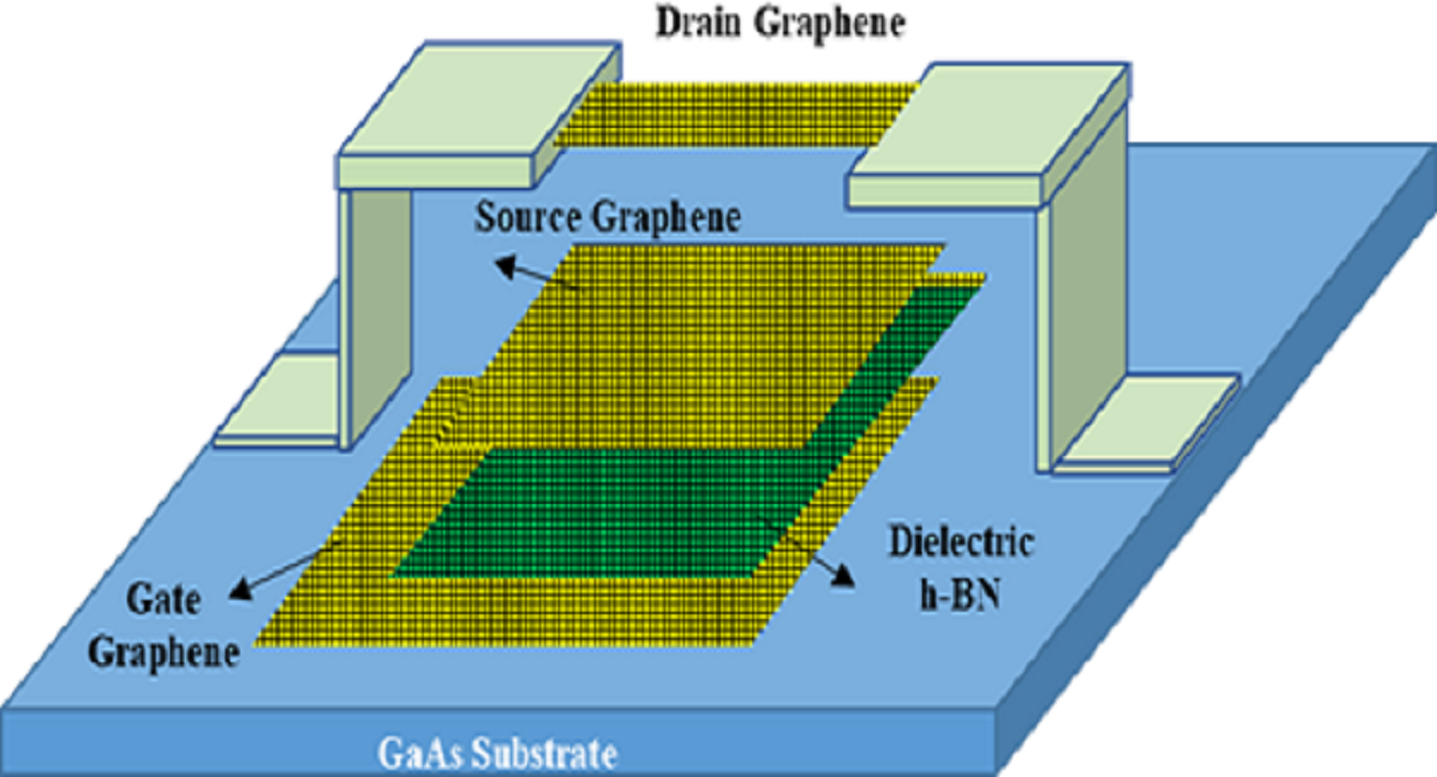
All flexible structure of a GR switch. Figure 4 was redrawn from [5].

In 2018, Huynh Van et al. [5] prepared a three-terminal fully flexible graphene switch for the first time, as shown in Figure 4. The switch is made of fully flexible materials. The three electrodes are all made of 1–2 layers of graphene, and the insulating medium layer is made of h-BN. The pull-in voltage at room temperature is less than 6 V, and the switch fails after 12 life cycles.

Graphene NEM switches can be used as phase shifters in RF MEMS systems due to the stable lattice and hydrophobic surface of graphene. In addition, GR-NEM switches can also be used as ESD devices in integrated circuits, which can greatly simplify the circuit volume of traditional PN junctions and increase the circuit integration.

#### NWs-NEM switches

Single-crystal nanowires are excellent candidates for NEM switches due to their uniform chemical and physical structure, and good reproducibility of structure and composition. Electrode materials include diamond, SiC, Si, and Ge. Table 3 summarizes nanowire materials commonly used for NEM switches.

**Table 3:** Structure and pull-in voltage of nanowire switches.

Date	Ref	Terminal	Material	Length [µm]	Gap [nm]	Voltage [V]

2010	[14]	two	Mo_6_S_3_I_6_	15	3000	21
2010	[36]	three	diamond	14	300	70
2010	[36]	three	diamond	14	100	5
2012	[37]	two	silicon	5	145	1.12
2015	[38]	three	silicon	1.8	50	1.8
2016	[39]	two	Bi_2_Se_3_	4	620	2.8
2019	[9]	two	Ge_0.91_Sn_0.09_	14.8	570	5
2021	[8]	two	CuO	3	120	12.5

The research on NWs-NEM switches can be classified into two types, namely manufacturing techniques and in situ techniques. In the former, the switches are first processed by top-down or bottom-up processes and, subsequently, their parameters are tested. In the latter, the pull-in effect of NWs is directly studied through atomic force microscopy or transmission electron microscopy using nanomanipulators. This allows one to explore different working states without having to manufacture multiple devices repeatedly.

**Figure 5:**
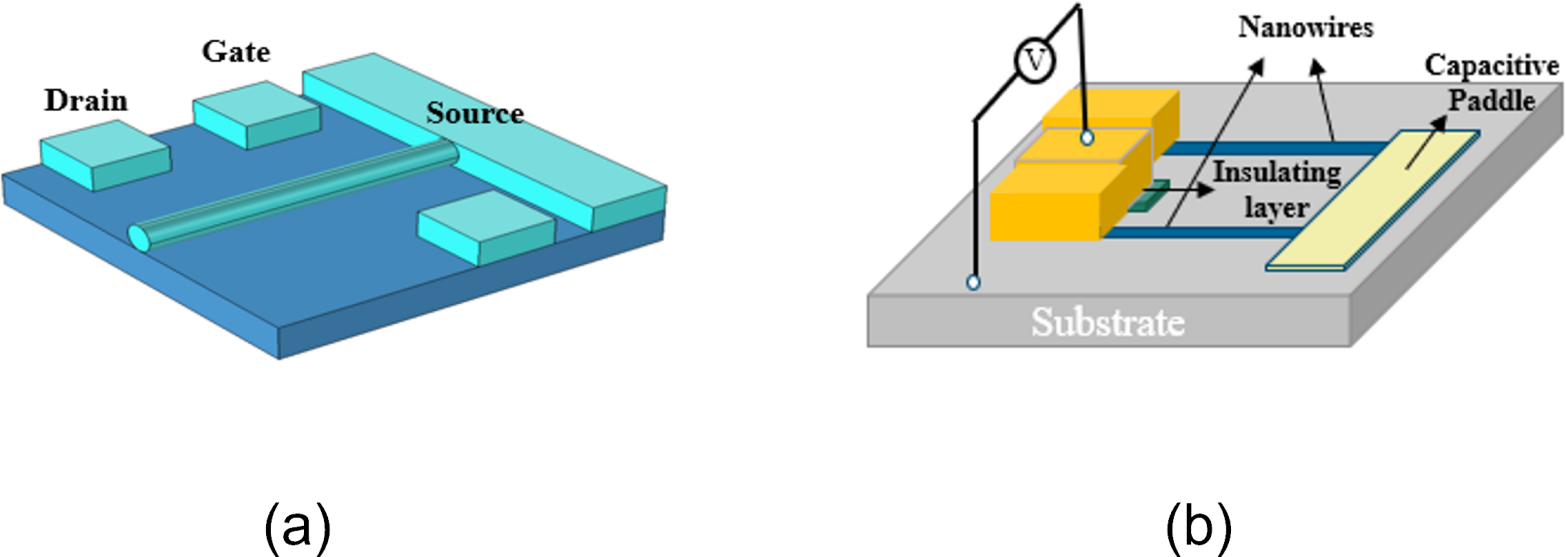
(a) Lateral structure and (b) U-shape structure of NW switches. Figure 5a,b were redrawn from [36–37], respectively.

**Manufacturing technique:** Liao et al. [36] first proposed the concept of a lateral switch structure in 2010, as shown in Figure 5a. The structure uses bilateral electrodes to assist recovery and improve lifetime. The flexible element uses a diamond NW as cantilever with cross section of 14 µm × 400 nm. The gap is 300 nm, and the pull-in voltage is 70 V. As the width is reduced to 200 nm and the gap is reduced to 100 nm, the pull-in voltage is reduced to 5 V. The switching current ratio is greater than 10^6^, and the number of life cycles is greater than 10^5^. Jasulaneca et al. [8] made a CuO NWs switch, 3 µm long, 80 nm in diameter, and 120 nm in the gap, with a pull-in voltage of 12.5 V. Feng et al. [40] prepared SiC nanowire NEM switches using bottom-up techniques. The pull-in voltage ranges from one to several volts and the response time is below microseconds with a switching ratio of about 10^3^.

Qian et al. [37] produced a U-shaped NEM switch with two Si nanowires, which support a square capacitive plate to form a U-shaped removable electrode, as shown in Figure 5b. The length of the silicon nanowires is 5 µm, the cross section is 90 × 90 nm square, the lateral gap is 2 µm, the gap between the electrode and the substrate is 145 nm, and the voltage is 1.12 V. However, the critical voltage fluctuation range is 1 V, and only five life cycles were achieved. Boodhoo et al. [38] used heavily doped Si NWs that were 1800 nm long, 42 nm wide and 50 nm thick, with a lateral spacing of 50 nm. The pull-in voltage was −1.8 V and the switching ratio was 10^5^. However, the introduction of heavy doping will increase the risk of failure.

**In situ technique:** In 2010, Andzane et al. [14] used the in situ technique to characterize Mo_6_S_3_I_6_ NW electrodes. The Mo_6_S_3_I_6_ beam was 15 µm in length and 3 µm in radius. The initial gap was 3 µm, and the pull-in voltage was over 21 V. In another publication [41], the authors used in the situ technique to glue Ge nanowires to electrochemically etched gold tips to form NEM switches. The pull-in voltage was 40–45 V with an initial electrode gap of 6 μm.

Kosmaca et al. [39] studied the electrostatic pull-in of Bi_2_Se_3_ NW electrodes and determined that the pull-in voltage was 3 V. This technique provides a new method for the connection between the intermediate layer of ITO and the electrode. In [10], they also used the in situ technique for resonance detection of a GeSn alloy. Meija et al. [42] proposed the technique of using resonance aid to reduce the pull-in voltage of Ge_0.91_Sn_0.09_ NWs. By applying 610.8 kHz and 0.45 V AC voltage to a GeSn alloy nanowire, the pull-in voltage can be reduced from 13.8 to 5 V. This method can effectively reduce the pull-in voltage as required by materials that are not resistant to higher voltages, such as Bi_2_Se_3_, and broaden the selection range of materials.

#### The influence of switch structure on the pull-in effect

The current NEM switches are still in the laboratory development stage, and the main obstacle to commercialization is the repeatability. Commercial switches require at least 10^15^ working cycles, but the current level is still 10^4^–10^8^. And the life cycles based on two-dimensional flexible materials such as graphene are only about a decade, far from meeting the needs of commercialization. NEM switches with low voltage and a high number of switching cycles are the main direction of research.

**Structure optimization:** The design of NEM switches with low voltage mainly considers the number of electrodes, mechanical structure, and driving mode. According to the number of electrodes, NEM switches can be divided into two-terminal, three-terminal, or multiterminal switches. The simple structure of the two-terminal switches requires fewer electrodes. Using additional electrodes for multiterminal switches reduces the elastic requirement for the materials and ensures a sufficiently large initial gap. According to the mechanical structure, NEM switches can be divided into cantilever (single clamp) and bridge (double clamp). Cantilevers are more sensitive with a lower critical voltage, while the stability of the beam is stronger when both ends are fixed. Samaali and Najar [43] proposed a double-beam RF switch, and the study showed that the pull-in voltage of the double-beam structure was 23% lower than that of the general structure. Prasanth et al. [44] designed structures with non-uniform shape and found that the pull-in voltage increased as the taper degree increased. Almitani et al. [45], Abdelrahman et al. [46], and Eltaher et al. [47–48] proposed a perforated-beam structure, based on cantilever and bridge structures. Their studies showed that the filling rate of holes significantly affected the resonant frequency of the beam. Guha et al. [49] and Sravani et al. [50] proposed a structure of perforated parallel plates supported by four serpentine springs. The elastic coefficient can be significantly reduced by the serpentine meander structure. Also, the capacitance and viscous air damping can be effectively reduced by the perforated structure, thus reducing the pull-in voltage.

**Lifetime extension:** Understanding the failure of the switch is the premise to improve the number of life cycles of switches. Mechanical tear, burn, and stiction are the main problems affecting the lifetime of NEM switches. A pressure of up to 30 GPa [51] when the switch is closed can easily cause wear and tear of the switches, especially for CNT [16] and GR [29] switches. Permanent adhesion caused by the dielectric charging [52–53] and chemical bonding [24] are the focus of the research on improving the life cycles. At present, it is mainly solved by reducing the electrode contact area [14] or increasing the electrode gap [54]. Due to the high voltage and the rapid local heating caused by the electrostatic discharge, the burning and melting electrode material will also affect the life cycles of the switch. Using AC voltage can reduce the need for high-temperature resistance of the electrodes. The performance of the switches can also be affected by the surrounding environment. Capillary forces are occurring due to the humid environment during wet etching [24]. In addition, the design of NEM switches needs to consider the unique characteristics of the materials. For example, the shape of CNTs not only affects the pull-in voltage, but also filled single-wall and bamboo-shaped MWCNTs show higher breaking fields than empty MWCNTs [55].

### Applications of NEM switches

NEM switches are basic components and are widely used in component-level and system-level applications. NEM switches can be used as low-power switches, phase shifters, and other basic components for RF communication. At the same time, there is extensive research on NEM switch structures in the preparation of microvalves, micropumps, or physical unclonable functions.

#### RF switches and phase shifters

Traditional RF MEMS systems use metal as the removable element to prepare RF switches. The response time of metal RF switches is about 1–15 µs, and the pull-in voltage is about tens of volts, which cannot meet the demand. Also, the metal lattice stability is poor. Electromigration or micro-welding adhesion failure might easily occur. GR-NEM switches with low power consumption can effectively reduce the pull-in voltage, improve the response speed, and overcome the shortcomings of metal switches, such as slow response and high critical voltage.

**Figure 6:**
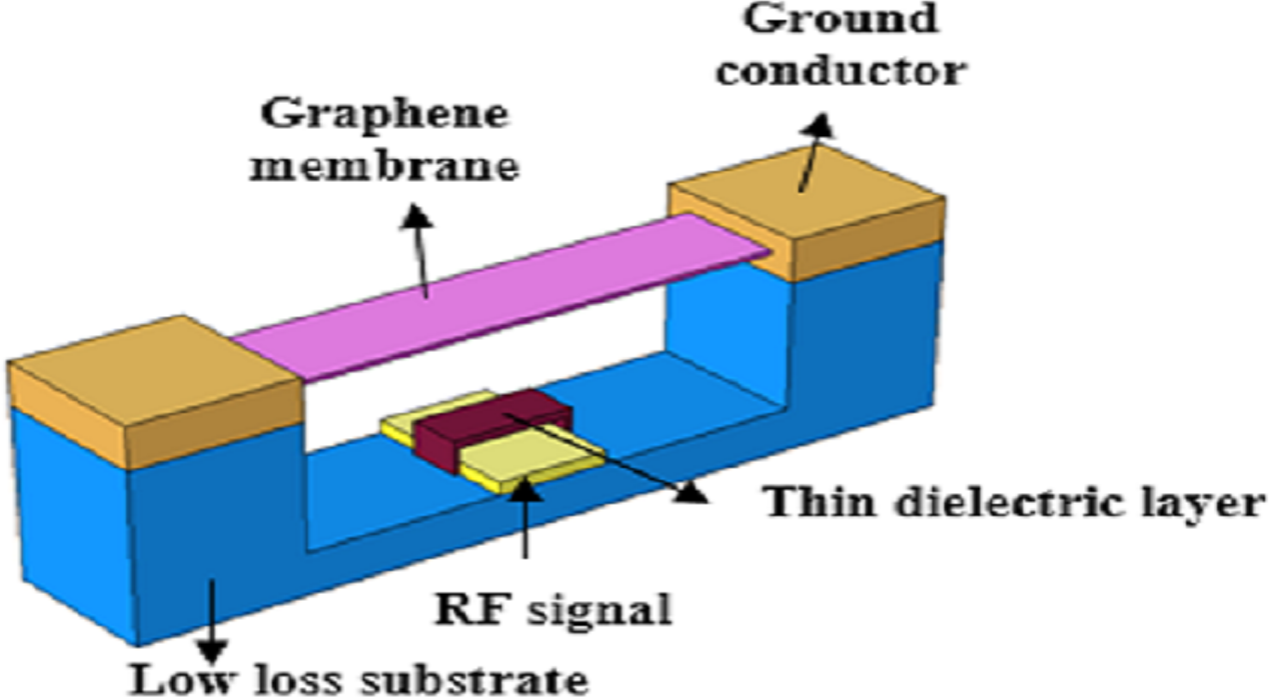
The structure of a GR-CPW phase shifter. Figure 6 was redrawn from [56].

Graphene NEM capacitor switches have been combined with coplanar waveguide (CPW) transmission lines as analog and digital phase shifters. Phase shifters are key components in smart antennas, and beam steering or scanning applications. The distributed microelectromechanical system transmission line (DMTL) is the most common type of phase shifter based on a load line structure. A DMTL consists of a CPW and a GR-NEM switching load. The GR-NEM switching load can affect the characteristic impedance of the transmission line, thus controlling the speed and generating a phase shift. A typical phase shifter structure is shown in Figure 6.

Dragoman et al. [57] first used a GR switch in a RF system. They deposited three gold bands onto a silicon substrate to form a CPW, and then prepared a suspended graphene bridge structure above the CPW with a pull-in voltage of 2 V and a wide operating frequency of 60 GHz. Sharma et al. [56,58] tested the isolation and insertion loss of graphene in microwave and millimeter-wave bands. At frequencies of 1–60 Ghz, the isolation of the single-layer graphene switch was 10 dB, and that of multilayer graphene was 20 dB. In addition, the isolation of the GR RF switch can be regulated by the applied voltage. Moldovan et al. [59] used multilayer graphene switches prepared by CVD for analog and digital bandwidth phase shifters. The phase shift was 355°/dB in the analog design at 2.4 GHz and 138°/dB in the digital design. Anjum et al. [60] used GR/MoS_2_ composite materials as beam to decrease the pull-in voltage below 1 V, which can be used in RF applications requiring low driving voltage.

#### ESD circuits

ESD protection is an indispensable part of integrated circuit (IC) design. Traditional in silico PN-junction-based on-chip ESD protection will cause parasitic capacitance, leakage, noise, a large consumption of silicon area, and other negative effects. This is unacceptable in the scale down of advanced IC designs. GR-NEM switches can be used as a new and unique ESD protection structure to replace PN junctions in integrated circuits. The high carrier mobility and the heat transfer coefficient allow for a low conduction resistance and can avoid device burn during ESD.

**Figure 7:**
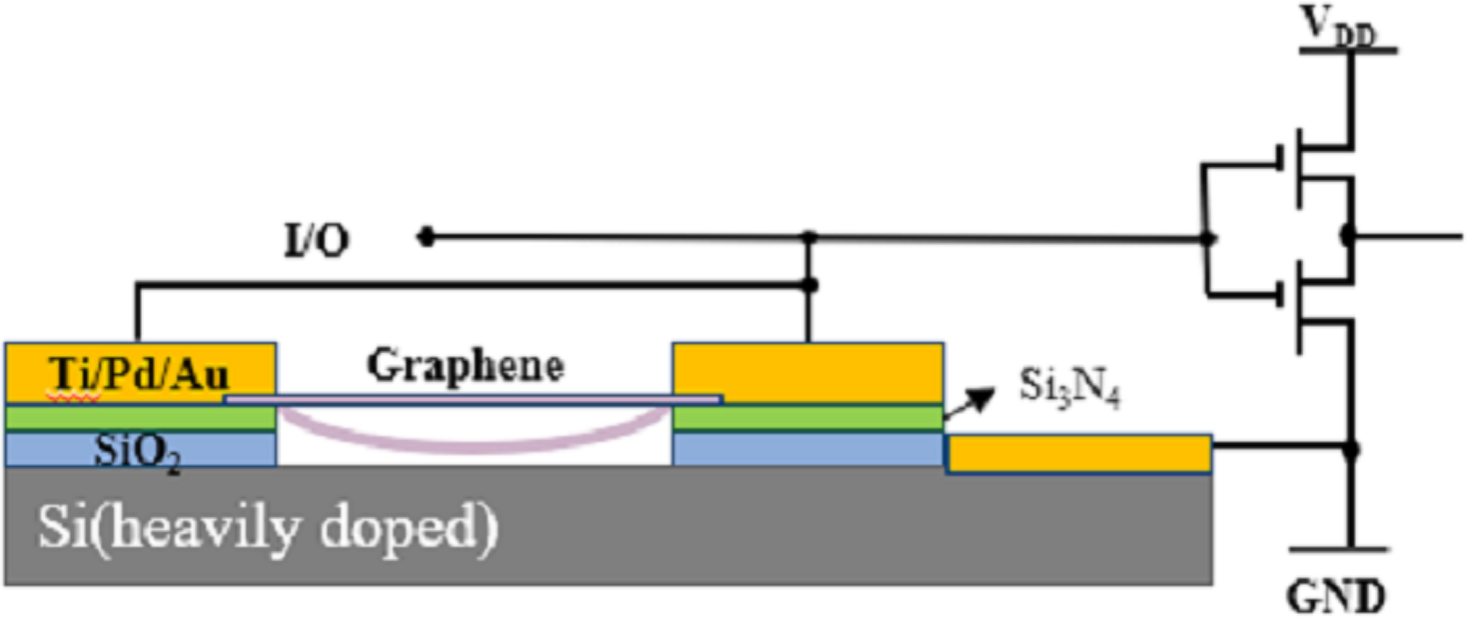
NEM switch used for ESD. Figure 7 was redrawn from [61].

ESD protection based on GR NEM switch structures can be integrated into the back end of the substrate, as shown in Figure 7. The anode and cathode of the switches are connected to input/output pads and ground pad on a chip, respectively. When electrostatic discharge occurs, the suspended graphene will be pulled into the bottom electrode, and the normally closed GR-NEM switch will be opened to release electrostatic discharge pulses.

Ma et al. [61] proposed that a model of an ESD circuit in which GR-NEM structures were used. The ESD voltage is 7–17.5 V, the response time is 200 ps, and the repetition cycle is more than 30 times. Chen et al. [62–64] tested GR-NEM ESD circuits using transmission line pulse (TLP) technology and assumed that transient ESD behavior was related to size and TLP waveform. Zhang et al. [65] explored the electrothermal characteristics of a Cu-GR interconnect under ESD, and the maximum temperature of the interconnect with a thickness of 10 nm could be reduced by 30%. Ng et al. [66] conducted stress tests on the prepared GR-NEM switch and proposed a nail structure to improve reliability. Li et al. [67–70] compared the effects of graphene materials on ESD performance. Due to the removal of grain boundaries, the single-crystal GR devices are four times more capable of handling current than polycrystalline GR devices. Shen et al. [71] prepared GR ESD switches and analyzed their electrical characteristics and reliability. The switch did work only once. Therefore, ESD protection based on the GR NEM switch structure needs to be further studied.

#### Microfluidic systems

**Figure 8:**
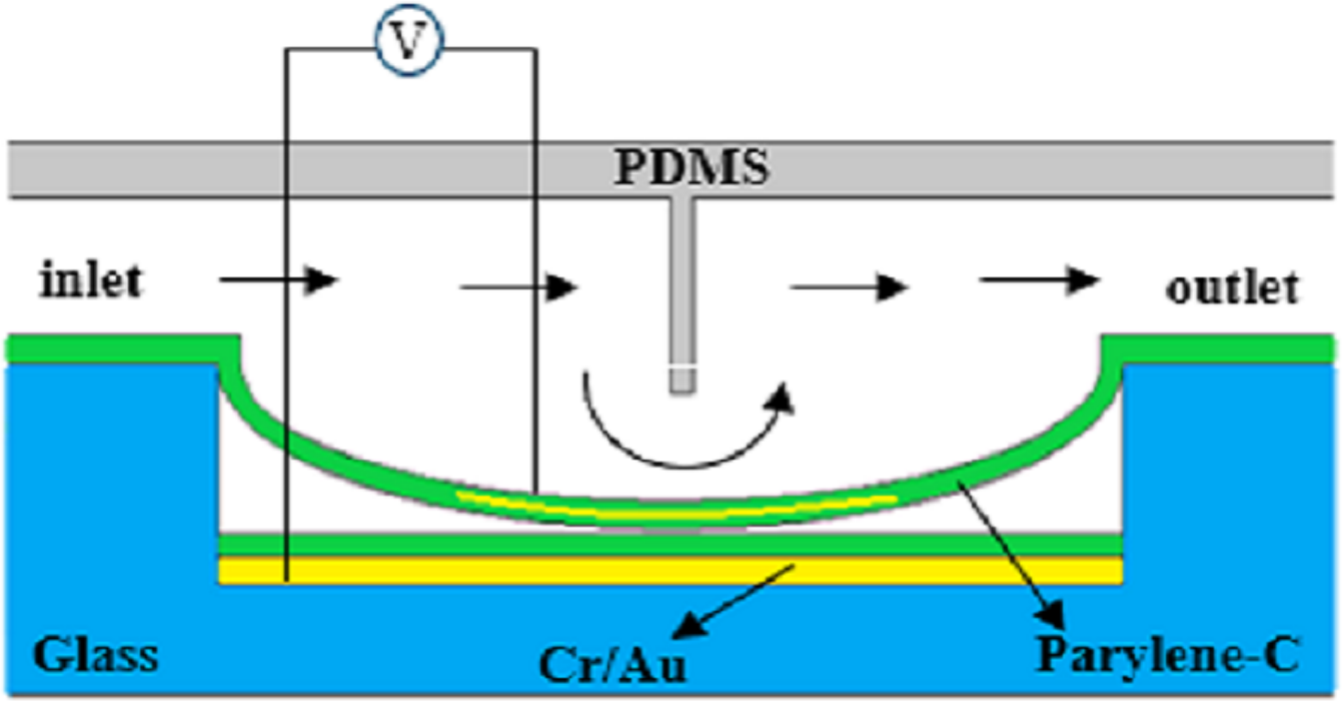
Schematic diagram of a flexible microvalve structure. Figure 8 was redrawn from [77].

Microfluidic systems composed of microvalves, microsensors, and micropumps are widely used in chemical engineering and life sciences. Electrostatically actuated microvalves reduce the required volume and are beneficial to the miniaturization of microfluidic systems. Electrostatically actuated microvalves contain a closing electrode, an opening electrode, and a flexible removable diaphragm. Bae et al. [72] fabricated a bidirectional microvalve using flexible polyimide as the valve diaphragm. The response time is 50 μs and can withstand 126 kPa pressure. Yıldırım et al. [73–74] used poly(*para*-xylene) to prepare a microvalve used in lab-on-chip devices. The pull-in voltage is 150 V and the valve can withstand pressure of 40 kPa. Desai et al. [75] and Patrascu et al. [76] designed a microvalve based on PDMS with a pull-in voltage of 150 V and a pressure capacity of 23 kPa and a life cycle of 400 times. Atik et al. [77] prepared a normally closed valve on glass substrates as shown in Figure 8. The valve required an average voltage of 221 V, the response time was less than microseconds, and the lifecycles was more than 50 times. The combination of flexible valves and PDMS microchannels optimizes the optical transparency and durability of microvalves.

**Figure 9:**
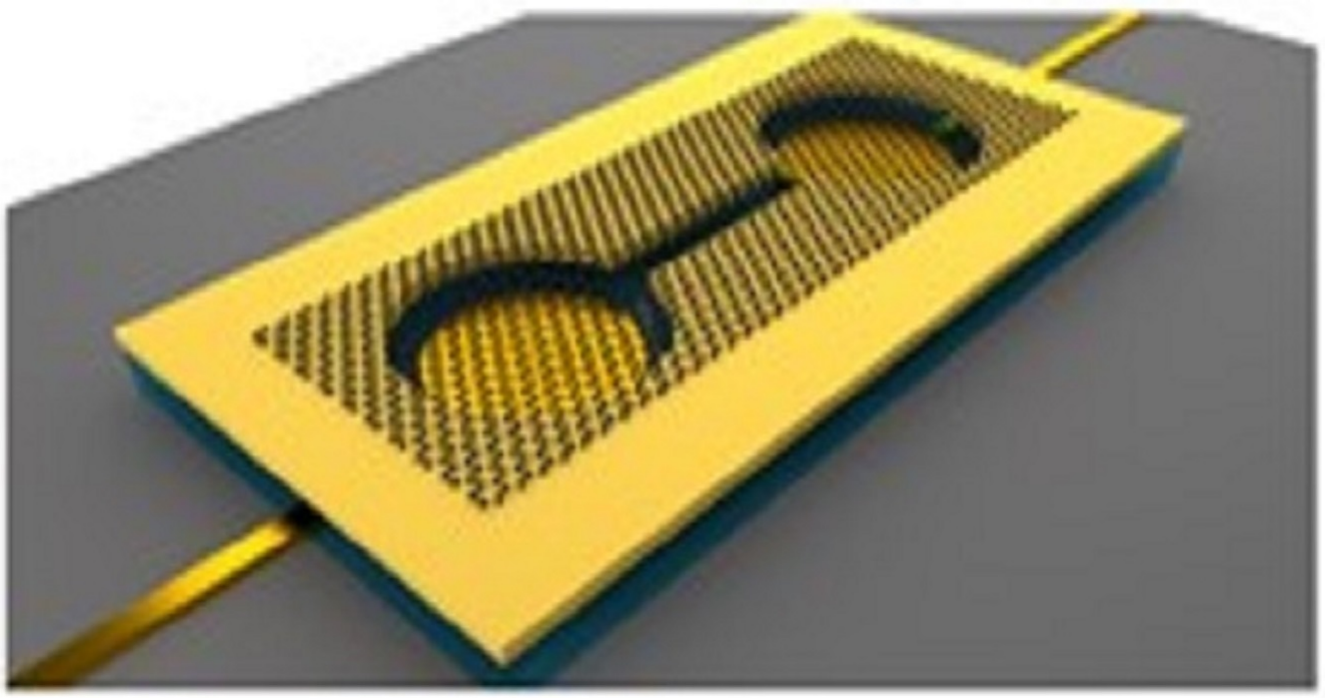
Schematic diagram of a GR pump. Figure 9 was republished from [78] (D. Davidovikj et al., “Graphene gas pumps”, 2D Materials, vol. 5, article no. 031009, published on 11 May 2018; https://doi.org/10.1088/2053-1583/aac0a8); © 2018 IOP Publishing. Reproduced with permission. All rights reserved. This content is not subject to CC BY 4.0.

A pneumatically coupled graphene membrane pump was designed by Davidovikj et al. [78], which is selectively permeable to gases in a controllable way, as shown in Figure 9. The dumbbell-shape chamber consists of a narrow trench connecting two circular cavities, both of which are covered by few-layer graphene. Local electrodes at the bottom of each cavity allow to drive each graphene membrane individually, changing the pressure of the chamber through a small voltage and controlling the airflow between the two cavities. The system can also be used for molecular sieving of gases and the aspiration and dispensing of liquids by introducing pores in the graphene membranes.

#### NEM physical unclonable functions

Just as NEM switches use the pull-in instability in microdevices as working principle, static stiction can be used in NEM switches as a mechanism to achieve a physical unclonable function (PUF) to ensure the reliability and stability of data. A PUF extracts the intrinsic physical parameters of a device and divides these parameters into the binary states “0” and “1”. Instead of storing encrypted information in digital memory, NEM-PUF creates a unique chip that takes advantage of the process differences inevitable in integrated circuit processing. Even though the mask and process are same, each chip is slightly different due to normal manufacturing variability.

**Figure 10:**
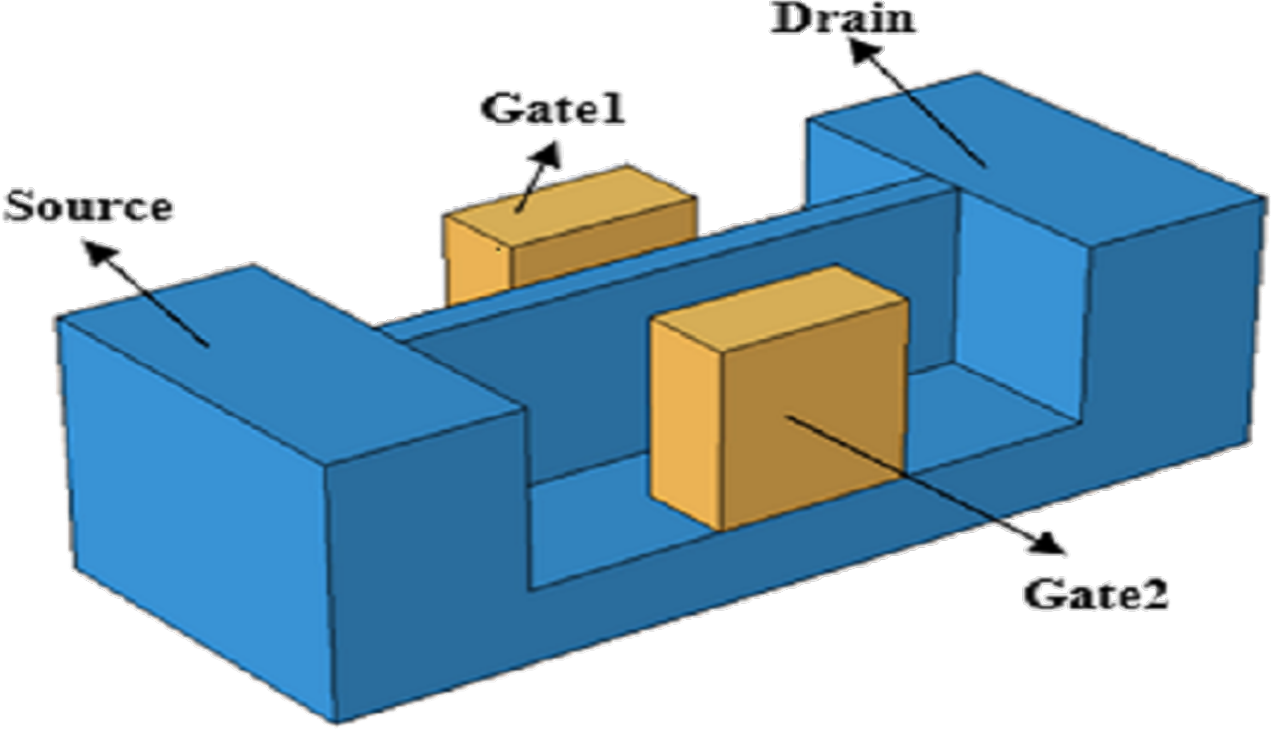
The structure of the NEM-PUF device. Figure 10 was redrawn from [79].

Boodhoo et al. [38] investigated the influence of the doping level on the gate voltage by preparing bilateral-gate Si NW transistors. Figure 10 shows the NEM-PUF structure prepared by Hwang and co-workers [79]. The device uses Si NWs as a moving electrode and the two gates (G1/G2) are symmetrically distributed. Si NWs in contact with G1 is defined as "0", and Si NWs in contact with G2 is defined as "1". The state of the PUF is determined by the current difference after G1/G2 conduction. In 2019, Hwang et al. [80] further improved the technique by increasing the states and expanding the encryption capacity by applying different doping levels on the source and drain sides of Si NWs. A NEM-PUF is ideal for outdoor and military applications due to the unique encryption formed by the random stiction of the devices during manufacturing.

#### Microscale energy systems

Electrostatically driven switches are known to require very high voltages. On the one hand, researchers are trying to optimize the size of the switches to find the optimal solution to reduce the voltage [81–84]. On the other hand, microswitches based on cantilever beam or bridge structures can be combined with triboelectric [85], pyroelectric [86], piezoelectric [87–89], and other power generation forms to form a microscale energy system. In this system, microswitches can be used as either rectifier to connect the power source to the load circuit or as an actuator structure, which plays an important role in the microscale energy system.

**Triboelectric power generation:** Two materials with different work functions will gain or lose electrons when they are in contact with each other, resulting in the triboelectric effect [90]. When the switch is closed, the electrode contact friction causes the surfaces to carry charges, forming a dielectric charging effect. Molinero et al. [52–53] characterized the dielectric charging when the switch electrodes were contacted and proved that the surface dielectric charging caused by friction would lead to a shift of the switch voltage and shorten the life cycles of the switch.

**Figure 11:**
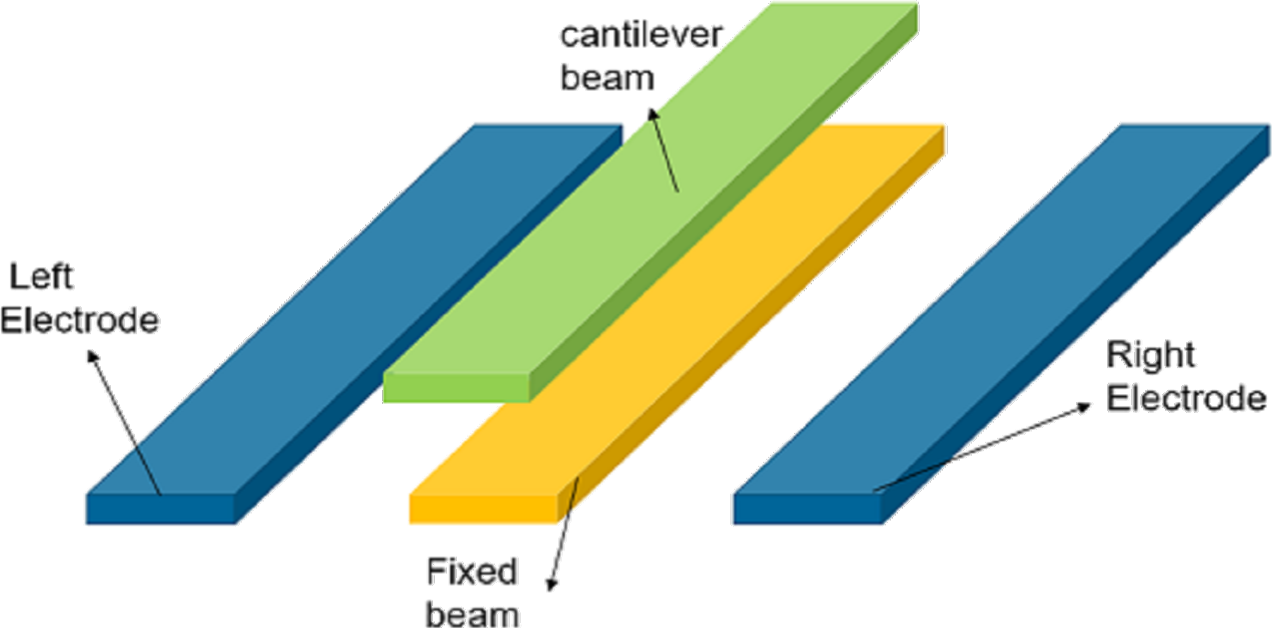
The structure of the sensor-switch system. Figure 11 was redrawn from [86].

Based on the coupling of triboelectrification and electrostatic induction, the switch can be used as a triboelectric generator (TEG) [90]. The TEG usually contains an insulated diaphragm and the switch is normally closed. The combination of triboelectrification and the elastic restoring force causes the switch to open. After the circuit conduction or charge disappears, the switch is pulled in again. By analyzing the electric field in the comb structure, He et al. [91] showed that the side electrode would generate a repulsive force without introducing a current. Pallay et al. [85] proposed a sensor–switch system as shown in Figure 11. The TEG consists of two aluminum electrodes and a PDMS-insulated diaphragm placed on a fixed electrode. When the TEG is impacted, the switch is opened with the help of the bottom side electrode. The TEG provides a way to solve the problem of high voltage of electrostatic switches. In addition, Cui et al. [92–95] showed that the self-powered triboelectrification nanogenerator (TENG) can also be used for human health detection and wound healing.

**Piezoelectric power generation:** Piezoelectric materials can produce very precise tiny motions and have significant characteristics of high force transmission [96], which is very suitable for MEMS applications. When the piezoelectric material is pressed, a potential difference will be generated between the two ends of the material. This potential difference can be used to assist the driving voltage of the NEM switch, thus reducing the need for external excitation.

**Figure 12:**
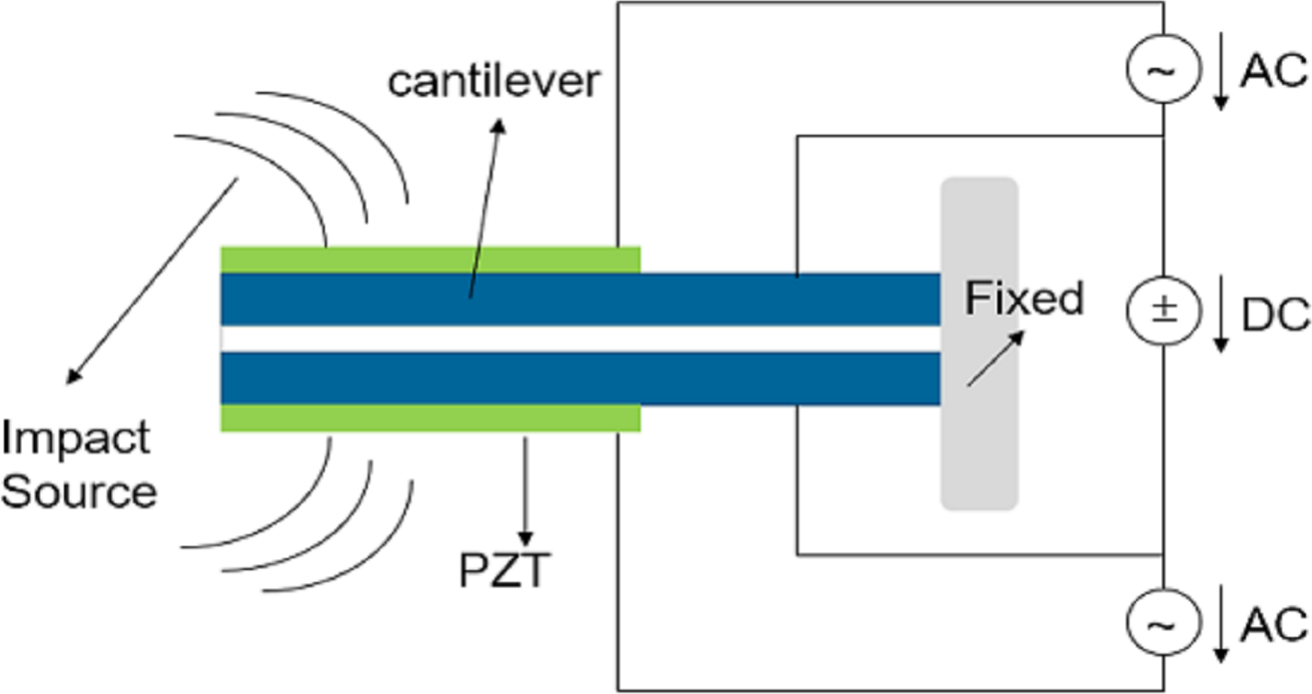
The structure of the plate transducer. Figure 12 was redrawn from [98].

Ultrasonic transducers based on capacitive switch structures are widely used in medical imaging, underwater tests, and obstacle detection. Saadatmand and Kook [97] conducted a theoretical analysis on electrostatically driven ultrasonic transducers with one-side and two-side circular plate structures and modified the voltage calculated by the lumped parameter model. Raeisifard et al. [96] proposed a comprehensive model of cantilever switches under electrostatic and piezoelectric excitation. They pointed out that piezoelectric excitation would reduce the demand for voltage of electrostatic switches, but would introduce additional manufacturing cost. Rutsch et al. [98] combined piezoelectric materials with NEM switches to prepare ultrasonic transducers, as shown in Figure 12. The structure retained piezoelectric bending and a DC voltage could be used to adjust the resonant frequency of the electrostatic switch to expand the bandwidth of the ultrasonic transducer. İkizoğlu et al. [99] combined a piezoelectric energy harvester with a cantilever beam switch for RF systems. Under vibrations, the piezoelectric material produces charges that are transferred via a switch rectifier to a capacitor to load it.

**Pyroelectric power generation:** Pyroelectric crystals can generate electricity with a change in temperature owing to the net change in polarization of the crystal units [100]. Therefore, it can be an ideal high-voltage source for MEMS/NEMS devices. Similar to the triboelectric effect, the pyroelectric effect can also be used as an energy source for microscale energy systems with NEM switches for corresponding sensors and actuators.

**Figure 13:**
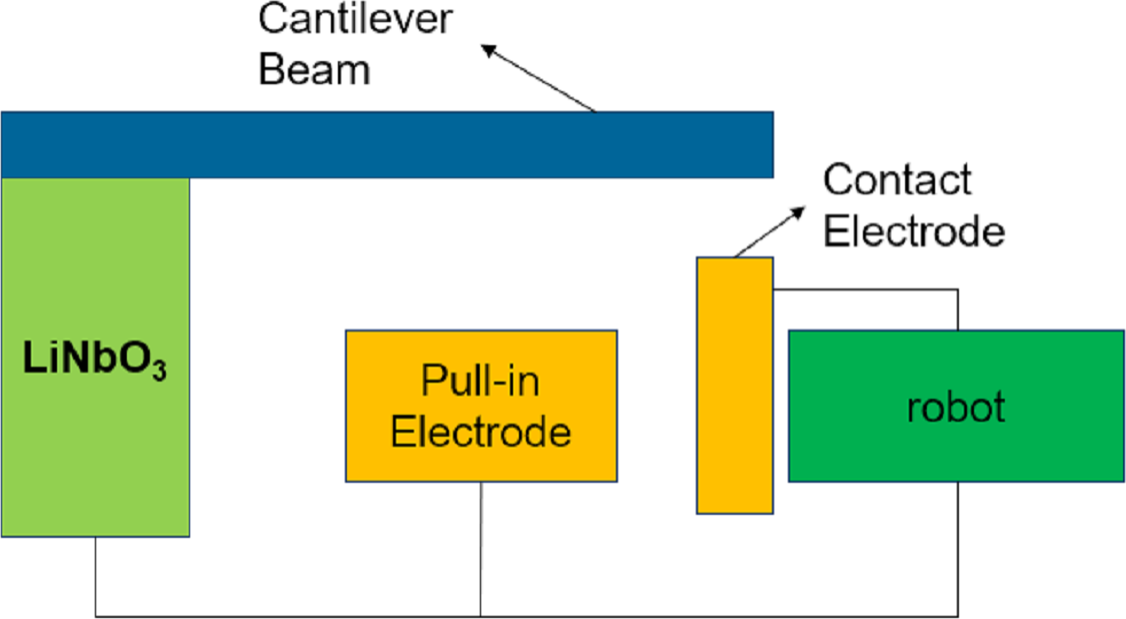
Structure of a PHVG system. Figure 13 was redrawn from [101].

Microscale robotics can be used to navigate and explore narrow spaces and manipulate microscale parts. A pyroelectric high-voltage generator (PHVG) system can produce voltages up to several kilovolts, which is an important power supply system for microscale robotics. Ni et al. [100] proposed a PHVG system composed of lithium niobate and a PZT cantilever switch, as shown in Figure 13. Lithium niobate can generate voltages with the help of a single-chip microcomputer containing heating and cooling functions. When the voltage exceeds the PZT cantilever switch pull-in voltage, the switch will be closed to complete the actuator task. In 2020, the authors [100] prepared a high-voltage source with 1.03 kV output voltage using 3D printed conductive polylactic acid polymer. In 2021, they [101] integrated a lithium niobate crystal, a piezoelectric cantilever switch, and a capacitive load into a single printed circuit board (PCB). The output voltage ranges from 15.5 to 1480 V, and the cantilever switch acts as a rectifier connected to PHVG to charge the capacitive load.

## Conclusion

The pull-in effect is a unique phenomenon in microscale and nanoscale devices, which is widely used in flexible devices. This review describes the preparation of NEM switches based on CNTs, GR, and NWs. By selecting material, structure, and other parameters of the flexible electrode, the performance of the switches can be improved. The effect has great potential in the fields of low-voltage applications and low power consumption. At the same time, as the most basic component, NEM switches can be used in RF systems, as ESD protection for integrated circuits, as microvalves and micropumps in microfluidic systems, and as PUFs to implement data encryption for outdoor and defense systems. In addition, NEM switches can also be combined with triboelectricity, pyroelectric, piezoelectric effects into microscale energy systems as sensors and actuators. However, most of the current research focuses on the use of flexible components. Also, there is some research on fully flexible devices, which still need further development.
